# Effect of *Lactobacillus kefiri*, in Conjunction with PENS T6 and a Hypocaloric Diet, on Weight Loss, Hypertension and Laboratory Glycemic and Lipid Profile

**DOI:** 10.3390/nu15214549

**Published:** 2023-10-26

**Authors:** Jaime Ruiz-Tovar, Carolina Llavero, Maria-Encarnacion Fernandez-Contreras

**Affiliations:** 1 EUEF San Juan de Dios, Universidad Pontificia de Comillas, 28036 Madrid, Spain; 2 Garcilaso Clinic, 28010 Madrid, Spain; carolinallavero@gmail.com; 3 School of Medicine, Universidad Alfonso X El Sabio, 28691 Madrid, Spain; mefdezcontreras@hotmail.com

**Keywords:** *Lactobacillus kefiri*, PENS T6, obesity, metabolic syndrome, dysbiosis

## Abstract

The pathogenesis of obesity has been linked to alterations in gut microorganisms. The aim of this study was to investigate the effect of *Lactobacillus kefiri*, together with PENS T6 and a hypocaloric diet, on weight loss, hypertension and laboratory glycemic and lipid profile. A prospective non-randomized study was conducted involving adult patients with a body mass index (BMI) > 30 kg/m^2^. Patients were divided into two groups: those undergoing PENS-T6 and hypocaloric diet (PENS-Diet Group) and those undergoing the same PENS-T6 scheme and hypocaloric diet, but additionally receiving probiotics including *Lactobacillus kefiri* (PENS-Diet + *L. kefiri* Group). Weight loss was assessed at the end of the treatment, and analytical glycemic and lipid profile, and microbiological analysis of feces were performed before and after treatment. The addition of *Lactobacillus kefiri* to PENS T6 and a low-calorie diet, increases weight loss and further improves the glycemic and lipid profile. *L. kefiri* also causes a further improvement in obesity-associated dysbiosis, mainly by increasing the muconutritive (*Akkermansia muciniphila*) and regulatory (*Bifidobacterium* spp.) microbiome, and the Phylum Bacteroidetes (*Prevotella* spp.) and decreasing the *Firmicutes*/*Bacteroidetes* ratio.

## 1. Introduction

More than half of the population in developed countries is overweight or obese to some degree. Obesity itself is a health risk factor that influences the development and progression of various diseases, such as dyslipidemia, ischemic heart disease, hypertension, type 2 diabetes mellitus, and sleep apnea-hypopnea syndrome, thus worsening patients’ quality of life, limiting their activities, and causing psychosocial problems. There is a direct relationship between body mass index (BMI) and morbidity and mortality risks in obese patients, which derives from associated pathologies and makes obesity itself a disease [1,2,3].

Dietary treatment associated with exercise is the first therapeutic step for tackling obesity. However, for it to be effective, patient motivation is essential, but often lacking. Obese patients often tire of following a low-calorie diet for long periods of time. A continuous feeling of hunger is the main cause of dietary treatment failure [1,4]. Percutaneous electroneurostimulation of the T6 dermatome (PENS T6) has been shown to reduce appetite and improve diet compliance, leading to significantly greater weight loss than hypocaloric diet alone and maintained for at least 1 year after treatment in patients with BMI > 30 kg/m^2^ [5,6,7].

The pathogenesis of obesity has been linked to alterations in gut microorganisms. The gut microbiota is composed of trillions of microorganisms, including at least 1000 known different bacterial species, located in the intestinal lumen or attached to the mucosal layer [8]. The five dominant bacterial phyla in the human gut are *Firmicutes*, *Bacteroidetes*, *Actinobacteria*, *Proteobacteria* and *Verrumicrobia* [9]. Regulatory bacteria are essential for local and systemic immunity, while the muconutritive microbiota are responsible for the formation of the mucus layer, and proteolytic bacteria have key metabolic functions in protein digestion [10]. The microbiota play different roles in the gut, such as in the metabolism of proteins, plant polyphenols, bile acids and vitamins, and in the assimilation of non-absorbable carbohydrates and short-chain fatty acids (SCFA) and gases.

Although the microbiota may show great variability within “healthy” individuals, obesity is associated with substantial changes in the composition and metabolic functions of the bacteria, resulting in an “obese microbiota”, which involves increased nutrient extraction from the diet [11,12]. One of the main characteristics of the “obese microbiota” is a lower prevalence of the phylum Bacteroidetes and a higher *Firmicutes*/*Bacteroidetes* ratio. However, the complexity behind how the gut microbiome modulates obesity may extend beyond a simple disproportion between these commensal phyla [13,14]. Different probiotics have been shown to balance the bacteria in the microbiota and thereby reduce body weight and metabolic and cardiovascular factors [15].

The aim of this study was to investigate the effect of *Lactobacillus kefiri*, together with PENS T6 and a hypocaloric diet, on weight loss, hypertension and laboratory glycemic and lipid profile.

## 2. Materials and Methods

A prospective non-randomized study was conducted in the Obesity Unit of Garcilaso Clinic (Madrid, Spain). Inclusion criteria were adult patients with a body mass index (BMI) > 30 kg/m^2^, with previous failure of dietary treatment. Exclusion criteria were untreated endocrine diseases causing obesity, portable electrical devices, and previous treatment with hormones, prebiotics, probiotics or nutritional supplements.

Patients were divided into 2 groups: those undergoing PENS-T6 and hypocaloric diet (PENS-Diet Group) and those undergoing the same PENS-T6 scheme and hypocaloric diet, but additionally receiving probiotics including *Lactobacillus kefiri* (PENS-Diet + *L. kefiri* Group). Weight loss was assessed at the end of the treatment, and analytical glycemic and lipid profile, and microbiological analysis of feces were performed before and after treatment.

### 2.1. Percutaneous Electrical Stimulation of Dermatome T6 (PENS)

PENS was performed as previously described [5,6,7] using the Urgent PC 200^®^ neuromodulation system (Uroplasty, Minnetonka, MN, USA). Patients were placed in the supine position and, without anesthesia, PENS was administered via a needle electrode inserted in the left upper quadrant along the mid-clavicular line, two centimeters below the rib cage, at a 90° angle to the abdominal wall and 0.5–1 cm deep. Successful insertion was confirmed by the sensation of electrical movement at least 5 cm beyond the dermatome territory. PENS was performed at a frequency of 20 Hz with the maximum amplification (0–20 mA) without causing pain. Participants underwent a 30-min session every week for 12 consecutive weeks.

### 2.2. Hypocaloric Diet

During the PENS interventions, a diet of 1200 Kcal/day was uniformly prescribed for both groups of patients, as previously published [5,6,7]. The diet followed a Mediterranean style (carbohydrate 51%, protein 23% and fat 26%) with a high consumption of fruits and vegetables, moderate consumption of meats and olive oil as the main source of fat [16]. We chose the Mediterranean diet, since it is the most popular in our environment and is based on foods that are easily available and consumed on a daily basis. In addition to weight loss during the treatment period, the aim is also to re-educate the patient’s diet, and for this it is necessary that the established diet is based on foods that can be consumed on a regular basis. We assume that the results in terms of weight loss could also be obtained with other types of diets, but we would lose the effect of dietary re-education if we did not include foods that are commonly consumed in the population studied. On the other hand, the Mediterranean diet is rich in fruits and vegetables, which are known prebiotics. Prebiotics will have a synergistic effect with probiotics in improving dysbiosis.

Throughout the study, food intake was recorded. Our dietician followed up via telephone to remind patients of the need to follow dietary recommendations and to resolve any eventual dietary problems.

No alcohol or nutritional supplements were allowed during the study.

### 2.3. Administration of Lactobacillus kefiri

Probiotic administration was based on the use of a commercial food supplement (Kefibios^®^, Hulka S.R.L., Rovigo, Italy), which contains live *Lactobacillus kefiri* (Lk) lactic ferments (LKF01-DSM 32079). The product is marketed in capsules. According to the product label, five drops of the solution reconstituted with 6 mL of vegetable oil in pre-filled vials contain ≥10^9^ active fluorescent units (AFU) of live and viable Lk. Patients received detailed instructions on how to use and mix the product, and the vial should be shaken before each administration. The product was then stored at room temperature between 10 °C and 25 °C and away from direct light. Five drops, corresponding to 10^9^ AFU, of the probiotic were administered once daily for 12 weeks.

### 2.4. Analysis of Microbiota

Fecal samples were obtained in OMNIgene-GUT tubes (Abyntek, Spain) at baseline and after treatments, and stored at −80 °C. Patients collected the sample themselves at home, following the manufacturer’s instructions. Samples were stored for 24 h at room temperature and then frozen (−80 °C) until use. The OMNIgene-GUT kit provides a valid method for preserving RNA at room temperature [17].

Total RNA was extracted from the feces (~50 mg) by dissolving it in Trizol reagent (Thermo Fisher, Madrid, Spain). RNA concentration and purity were assessed by 260/280 nm ratio using the Nanodrop spectrophotometer (Nirko). Equal amounts of RNA were reverse transcribed to obtain cDNA for quantitative PCR (qPCR). Gene expression assays were labelled with the Fam fluorophore, while the housekeeping gene was labelled with VIC fluorophore. Amplification conditions were 2′ at 50 °C, 10″ at 95 °C and 40 cycles of 15″ at 95 °C and 1′ at 60 °C (AB7500 fast and Quant Studio 5; Thermo Fisher). All samples were prepared in triplicate to obtain their threshold cycle (Ct). If the deviation for each triplicate was greater than 0.3 cycles, the Ct was not considered. The relative expression of each gene was obtained following the model R = 2^−ΔΔΔCt^. The primer was designed for the ribosomal RNA (16S) genes of the main bacterial groups present in the mammalian gut microbiota, including *Firmicutes*, *Bacteroidetes*, *Proteobacteria*, *Fusobacteria*, *Actinobacteria* and *Verrucomicrobia* [18]. To determine bacterial composition, we used bacterial species-specific primers. The specificity of the primers was tested in silico with the probe match tool of the Ribosomal Database Project (http://rdp.cme.msu.edu/ accessed on 15 June 2022), and validated in the BLAST search (NCBI) [19]. Primers were purchased from Thermo Scientific and stored at −20 °C.

Reference ranges for gut bacteria were calculated as the mean gene copy number (GCN) of fecal samples from a control population of volunteer patients. Fecal samples from 100 non-obese, normoglycemic and normolipidemic age- and sex-matched volunteers without known cardiovascular, malignant and digestive diseases were analyzed to estimate control ranges for each bacterium.

### 2.5. Analytical Variables

Blood samples were centrifuged for 20 min at 2500× *g* and the plasma obtained was analyzed for glucose and lipids. Fasting glucose and glycosylated hemoglobin (A1C), as well as lipid profile (triglycerides, total cholesterol and HDL-cholesterol) were quantified by standard methods (ADVIA 2400 Chemistry System, Siemens, Germany). All variables were measured before and after the interventions.

### 2.6. Clinical Variables

Anthropometric parameters at baseline and after interventions included body mass index (BMI) and weight loss. Systolic and diastolic blood pressure was assessed with an automatic blood pressure monitor (Omron M2-HEM-7121-E, Kyoto, Japan).

Stool consistency was established using the Bristol Stool Scale, and was determined both at the beginning and at the end of the assigned treatment [20].

### 2.7. Statistical Analysis

Quantitative variables were defined by mean values and standard deviation, or by median and range, depending on the Gaussian distribution. Normally distributed variables were compared via Student’s *t*-test for independent and paired samples, whereas non-normally distributed variables were compared via the Mann–Whitney U test for independent samples, and a Wilcoxon signed-rank test for paired samples. Values of *p* < 0.05 were considered significant. Statistical analyses were performed with the statistical package for social sciences (SPSS, IBM, Armonk, NY, USA), version 28.0.

## 3. Results

A total of 60 patients were included, 19 males (31.7%) and 41 females (68.3%), with a mean age of 44.3 +/− 8.1 years. At baseline, mean weight was 87.9 +/− 8.1 kg (range 76–106.8 kg) and median body mass index (BMI) 32.8 +/− 4.6 kg/m^2^ (range 30.2–45.6 kg/m^2^). Mean blood pressure levels and glycemic and lipid profile parameters were within normal range. There were no significant differences between the groups. Mean weight was significantly higher in the PENS-Diet + *L. kefiri* group, but this difference was not reflected in the mean BMI (Table 1).

### 3.1. Evolution of Weight Loss, Blood Pressure, and Glycemic and Lipid Profiles

After 12 weeks of treatment, patients presented a reduction in BMI to 27.6 +/− 3.4 kg/m^2^, with a mean weight loss of 14.0 +/− 5.3 kg and a mean total weight loss (TWL) of 15.6 +/− 4.7%.

Systolic blood pressure showed a mean reduction of 12.5 +/− 10.0 mmHg and diastolic blood pressure of 11.0 +/− 16.1 mmHg. However, there were no cases of hypotension in either group.

Glucose levels showed a mean reduction of 23.4 +/− 8.9 mg/dL with a mean A1C reduction of 0.3 +/− 0.5%. Similarly, triglyceride values showed a mean decrease of 39.3 +/− 33.6 mg/dL and total cholesterol of 10.3 +/− 27.7 mg/dL, while HDL-cholesterol values showed an increase of 4.3 +/− 12.6 mg/dL. The differences between groups are shown in Table 2.

Significantly greater weight loss and improved glycemic parameters were observed in the PENS-Diet + *L. kefiri* group. Regarding lipid profile, triglyceride reduction and HDL-cholesterol increase were significantly higher in the PENS-Diet + *L. kefiri* group.

When baseline values were compared with post-procedure values in the PENS-Diet + *L. kefiri* group, significant reductions were observed in systolic and diastolic blood pressure, fasting glucose, A1c, triglycerides and total cholesterol levels, while there was a significant increase in HDL-cholesterol values (Table 3). Similar results were obtained in the PENS-Diet group, but without reaching statistically significant differences in A1c and total cholesterol (Table 4).

### 3.2. Evolution of Gut Microbiota

There were no significant differences between the groups in the baseline distribution of gut microbiota. Overall, the muconutritive microbiota was below the reference range. The two phyla, *Firmicutes* and *Bacteroidetes*, were also below the reference range, with a significantly increased *Firmicutes*/*Bacteroidetes* ratio. The phyla *Actinobacteria* and *Verrucomicrobia* were also below the reference range (Table 5).

After the exclusive application of a hypocaloric diet and PENS, a significant increase in the muconutritive microbiota, mainly *Akkermansia muciniphila*, was observed. Similarly, a significant increase in Lactobacillus was also determined, although no significant differences in the regulatory microbiota could be established (Table 6).

In the PENS-Diet + *L. kefiri* group, significant increases in the muconutritive and regulatory microbiota were observed, mainly secondary to increases in *Akkermansia muciniphila* and *Lactobacillus* spp. In addition, a significant decrease in the phylum *Firmicutes* and a significant increase in the phylum Bacteroidetes were determined. Consequently, The *Firmicutes*/*Bacteroidetes* ratio showed a significant reduction. Within the phylum *Bacteroidetes*, both species, *Prevotella* spp. and *Bacteroides* spp. presented a significant increase, although the increase in *Prevotella* spp. values was especially relevant. In addition, the phylum Proteobacteria also showed a significant reduction (Table 7).

Significant increases in muconutritive microbiota, *Akkermansia muciniphila* and *Lactobacillus* spp. were observed in both groups. However, when analyzing the increases separately, it can be determined that the increase was significantly higher in the PENS-Diet + *L. kefiri* group (Table 8).

### 3.3. Bristol Stools Scale

According to the Bristol stools scale, the median baseline values received a score of 5, with a range between 3 and 5. There were no significant differences between groups.

In the PENS-Diet group, the median values received a score of 2 (range 1–4), representing a significant trend towards constipation (*p* < 0.001). However, in the PENS-Diet + *L. kefiri* the values remained at a median score of 4 (range 2–5) (*p* = 0.450).

In the PENS-Diet group two patients scored their stool with a 1, meaning severe constipation. In both cases, laxatives had to be prescribed despite the high content of fruits and vegetables in the prescribed Mediterranean diet. There were no cases of severe constipation in the PENS-Diet + *L. kefiri* group.

## 4. Discussion

PENS T6 for the treatment of obesity was initially described by our group in 2014. It was initially applied to morbidly obese patients awaiting bariatric surgery, with the aim of reducing weight before surgery and consequently reducing surgical risk. In addition, patients who underwent PENS T6 showed complete adherence to the diet in more than 95% of cases and significantly reduced feelings of hunger. Given these results, the indication for PENS T6 was extended to patients classified as overweight and mild-to-moderate obesity, revealing an average weight loss of more than 10 kg in 10 weeks and maintained for at least 1 year after the end of the therapy. The basis of weight loss is the low diet abandonment, due to the absence or low sensation of hunger. Ghrelin is an orexigenic hormone, released by the gastric fundus in response to the perception of an empty stomach and which acts on the appetite center in the central nervous system, causing a sensation of hunger. The PENS of the T6 dermatome triggers a somato-autonomic reflex with gastric stimulation via the vagus nerve. As a result, gastric emptying is slowed down, and as food remains longer in the stomach, ghrelin secretion is inhibited, thus reducing the sensation of hunger and allowing greater adherence to low-calorie diets. In our previous studies, we have demonstrated a significant decrease in ghrelin levels in patients undergoing PENS T6 [5,6,7].

As described in our previous publications, the patients in the present study undergoing PENS T6 associated with diet, showed significant weight loss, reduction in systolic and diastolic blood pressure values, and fasting glucose and triglyceride levels. These effects could be justified by the caloric restriction and by the neurostimulation of the gastric wall and promotion of early satiety [7]. In addition, the implication of a reduction in insulin resistance mediated by a decrease in counter-regulatory hormones has also been hypothesized [21].

However, significantly greater improvement in both glycemic and lipid profile, as well as greater weight loss, was observed among patients with *Lactobacillus kefiri* intake. The etiopathogenesis of obesity and its associated comorbidities is multifactorial. Increasing evidence presented in the literature suggests the involvement of gut dysbiosis in the development of obesity [22]. Baseline analysis of microbiota revealed dysbiosis, with a reduction in muconutritive bacteria such as *Akkermansia muciniphila*, *Faecalibacterium* spp., and *Bifidobacterium* spp. In addition, the *Firmicutes*/*Bacteroidetes* ratio was significantly elevated. It is still unclear whether these variations in the microbiota may have determined the development of obesity or whether they are a consequence of it [23,24].

After the intervention, a significant increase in muconutritive bacteria, especially *Akkermansia muciniphila*, in addition to *Lactobacillus* spp., and a significant decrease in the *Firmicutes*/*Bacteroidetes* ratio were observed in the PENS-diet group. The improvement in this ratio has often been related to weight loss and intestinal inflammation and permeabilization [25].

However, in the PENS-Diet + *L. kefiri* group the increase in muconutritive bacteria, *Akkermansia muciniphila*, and *Lactobacillus* spp., and the decrease in the *Firmicutes*/*Bacteroidetes* ratio were even greater, with significant differences between groups. Furthermore, the addition of *Lactobacillus kefiri* also resulted in a significant increase in regulatory microbiota in general (not only *Lactobacillus* spp.) and in the phylum *Bacteroidetes* (both *Bacteroides* spp. and *Prevotella* spp., especially the latter), with a significant decrease in the phylum *Firmicutes* and, consequently, a further decrease in the *Firmicutes*/*Bacteroidetes* ratio. 

*Prevotella* spp. has demonstrated to induce beneficial effects on mucin regulation, glucose metabolism and hepatic glycogen storage [26]. *Bifidobacterium* spp. has been shown to be beneficial for gastrointestinal barrier function and immunoregulation [27]. By increasing the abundance of *Bifidobacterium* spp., intestinal permeability has been reduced in obese mice, which correlates with a decrease in inflammatory markers [28]. Furthermore, *Bifidobacterium* spp. produce lactate, which is transformed into butyrate by butyrate-producing bacteria in the intestine [29]. These short-chain fatty acids (SCFA) play a protective role against cardiovascular risk by regulating lipid and glucose metabolism, and producing glucagon-like peptide-1, peptide YY and leptin [30]. In addition, butyrate induces mucin synthesis and protects gut integrity. *Akkermansia muciniphila* also regulates intestinal permeability [31]. Its abundance has been inversely correlated with adipose tissue inflammation and insulin resistance in mice and humans [32,33]. In hyperlipidemic obese mice, *Akkermansia muciniphila* also improved metabolic endotoxemia, vascular inflammation and atherosclerotic lesions [34]. Taken together, the enrichment of muconutritive and regulatory bacteria observed in our patients could also participate in the improvement of their plasma metabolic and cardiovascular factors, as well as in the attenuation of their body weight.

Literature data have shown that mono- or multi-strain probiotics alone produce minimal changes in body weight and glycemic or lipid profiles; their benefit is concurrently associated with diet [35]. Moreover, single probiotics, such as *Lactobacillus gasseri*, *Bifidobacterium animalis* and *Pediococcus pentosaceus* have been shown to achieve greater benefits than multiple probiotics (combinations of *Bifidobacterium* spp., *Lactobacillus* spp. and/or *Lactococcus* spp.). A previous study of our group, in which PENS was combined with a hypocaloric diet and a combination of *Lactobacillus plantarum LP115*, *Bifidobacterium brevis B3*, and *Lactobacillus acidophilus LA14*, also observed a positive influence on anti-obesogenic gut bacteria by increasing muconutritive (*Akkermansia muciniphila*) and regulatory (*Bifidobacterium* spp.) microbiota, and phylum *Bacteroidetes* (*Prevotella* spp.), and a reduction in *Firmicutes*/*Bacteroidetes* ratio; clinically, they induced a further reduction in body weight and plasma A1C, triglycerides, and HDL-cholesterol [36].

Previous studies have demonstrated that the administration of *Lactobacillus kefiri* as a single probiotic in mice downregulates the expression of proinflammatory mediators and increases anti-inflammatory molecules in the gut immune system. In humans, it also regulates intestinal homeostasis, incrementing immunoglobulin A secretion [37,38,39]. There is still little evidence regarding the changes in bacterial phyla and species after the administration of *Lactobacillus kefiri* as a single probiotic. Further studies must be conducted to confirm our results.

Despite the Mediterranean diet being rich in fruits and vegetables, given that in low-calorie diets the overall food intake is reduced, this leads to a reduction in the fecal bolus and therefore the appearance of constipation is frequent. This is what occurred in our PENS-Diet group. However, in the PENS-Diet + *L. kefiri* group, intestinal transit was maintained with stool production within the normal range (median Bristol score 4). There is no evidence in the literature justifying the addition of *Lactobacillus kefiri* to prevent constipation. However, the addition of *Lactobacillus kefiranofaciens*, a microorganism of the same family and bearing great similarity with the *Lactobacillus kefiri*, has demonstrated to induce higher total fecal weight and higher fecal water content in mice. Furthermore, *Lactobacillus kefiranofaciens* in this study also provoked the increasement of *Firmicutes*, *Bacteroidetes*, *Lactobacillus*, and *Prevotella*. Consequently, the prevention of constipation that we observed in our study can also derive from changes in the gut microbiota [40].

### Limitations

The main limitation of the present study was a lack of randomization. Although the initial idea was to make the study randomized, given that it was impossible to make it blind for the patient, they knew beforehand about the benefits of probiotics and refused to participate in the randomization. Therefore, the inclusion of patients in the control group (without probiotics) was based on instances where probiotics were not available since we depended on their free supply by Hulka SRL. Despite this drawback, as we have seen in the results, there were few significant differences between the groups in clinical variables and no significant differences in the composition of the microbiota. Baseline weight was significantly higher in the PENS-Diet + *L. kefiri* group, but no significant differences in baseline BMI could be determined. It seems clear that a greater weight reduction could be obtained in the heavier group. However, after treatment, the PENS-Diet + *L. kefiri* group showed not only a significantly greater weight loss, but also a greater mean reduction in BMI and total weight loss. However, this difference in baseline weight could be considered a bias. Future studies should match all baseline characteristics between groups.

Rapid weight loss, as in the present study, is often accompanied by several negative effects such as insomnia and eating disorders. It would have been interesting to investigate these factors, to assess whether the addition of probiotics could have mitigated them to some extent.

Other factors that could influence the results and thus warranting consideration include unknown comorbidities or habits that might alter bacterial distribution and probiotic action. Furthermore, physical activity could have also influenced weight loss and analytical glycemic and lipid profiles. Despite all the patients receiving the same recommendations to perform moderate physical activity for at least 1 h daily, this parameter was not monitored in the study. Furthermore, changes in glycemic and lipid profiles could be influenced by fluctuations in insulin levels and changes in insulin resistance. Further studies must be conducted evaluating both insulin levels and insulin resistance.

Finally, we must accept the evaluation of the gut microbiota based on PCR amplification of the V3–V4 regions of their respective 16S rRNA genes as a more accurate method to analyze dysbiosis. However, these analyses are more expensive, and the lack of sufficient funding forced us to carry out the current analysis. Future studies must confirm our results with PCR analysis.

## 5. Conclusions

The addition of *Lactobacillus kefiri* to a hypocaloric diet and coadjuvant methods, like PENS T6, increases weight loss and further improves glycemic and lipid profiles in participants. *L. Kefiri* also provokes a further improvement in the dysbiosis associated with obesity, mainly increasing muconutritive (*Akkermansia muciniphila*) and regulatory (*Bifidobacterium* spp.) microbioma, and Phylum Bacteroidetes (*Prevotella* spp.) and decreasing the *Firmicutes*/*Bacteroidetes* ratio.

## Figures and Tables

**Table 1:** Distribution of age, gender, and baseline anthropometric measurements, blood pressure, glycemic and lipid profile between groups.

	PENS-Diet (N = 30)	PENS-Diet + *L. kefiri* (N = 30)	*p*
Age (years)	44.2 +/− 9.2	44.3 +/− 7.0	0.963
Females/Males	20/10	21/9	0.781
Weight (kg)	84.8 +/− 4.8	91.1 +/− 9.5	0.002
BMI (kg/m^2^)	32.3 +/− 3.5 (range 30.8–44.8)	33.4 +/− 3.5 (range 30.2–45.6)	0.392
Systolic blood pressure (mmHg)	135.5 +/− 11.6	131 +/− 12.7	0.157
Diastolic blood pressure (mmHg)	81.5 +/− 9.4	82.5 +/− 6.0	0.625
Fasting glucose (mg/dL)	101.4 +/− 26.0	100.1 +/− 12.4	0.814
A1c (%)	5.4 +/− 0.8	5.6 +/− 0.6	0.537
Triglycerides (mg/dL)	151.2 +/− 60.1	152.5 +/− 36.6	0.918
Total cholesterol (mg/dL)	201.3 +/− 49.2	191.7 +/− 34.0	0.383
HDL-cholesterol (mg/dL)	53.8 +/− 17.7	48.7 +/− 12.4	0.219

The chi-square test was used to compare sexes. For quantitative variables, the Student’s *t*-test was used when the variables followed a Gaussian distribution. The Mann–Whitney test was used to compare non-Gaussian variables.

**Table 2:** Post-intervention differences in weight loss, blood pressure, and plasma parameters between PENS-Diet and PENS-Diet + *L. kefiri*.

	PENS-Diet (N = 30)	PENS-Diet + *L. kefiri* (N = 30)	*p*
Weight loss (kg)	11.2 +/− 4.3	16.7 +/− 4.6	<0.001
BMI reduction (kg/m^2^)	4.3 +/− 1.8	6.2 +/− 1.9	<0.001
Total weight loss (%)	13.0 +/− 4.4	18.2 +/− 3.5	<0.001
Difference in Systolic blood pressure (mmHg)	14.5 +/− 12.4	10.5 +/− 6.6	0.126
Difference in Diastolic blood pressure (mmHg)	11.8 +/− 10.5	10.2 +/− 20.7	0.702
Difference in fasting glucose (mg/dL)	8.5 +/− 8.9	15.5 +/− 12.8	0.019
Difference in A1c (%)	0.2 +/− 0.6	0.5 +/− 0.4	0.030
Difference in Triglycerides (mg/dL)	16.5 +/− 13.1	59.9 +/− 33.3	<0.001
Difference in total cholesterol (mg/dL)	5.4 +/− 19.5	15.2 +/− 33.6	0.174
Difference in HDL-cholesterol (mg/dL)	1.1 +/− 6.4	−9.1 +/− (−14.9)	0.01

For quantitative variables, the Student´s *t*-test was used when the variables followed a Gaussian distribution. The Mann–Whitney test was used to compare non-Gaussian variables.

**Table 3:** Evolution of blood pressure, glycemic and lipid profiles in the PENS-Diet + *L. kefiri* group.

	Baseline	Postprocedure	*p*
Systolic blood pressure (mmHg)	131 +/− 12.7	120.5 +/− 8.2	<0.001
Diastolic blood pressure (mmHg)	82.3 +/− 6.0	72.3 +/− 4.7	0.012
Fasting glucose (mg/dL)	100.1 +/− 12.4	84.6 +/− 6.9	<0.001
A1c (%)	5.6 +/− 0.6	5.1 +/− 0.4	<0.001
Triglycerides (mg/dL)	125.5 +/− 36.6	92.6 +/− 32.7	<0.001
Total cholesterol (mg/dL)	191.7 +/− 34.0	176.5 +/− 45.6	0.019
HDL-cholesterol (mg/dL)	48.7 +/− 12.4	57.8 +/− 13.3	0.002

For quantitative variables, the Student´s *t*-test was used when the variables followed a Gaussian distribution. The Mann–Whitney test was used to compare non-Gaussian variables.

**Table 4:** Evolution of blood pressure, glycemic and lipid profiles in the PENS-Diet group.

	Baseline	Postprocedure	*p*
Systolic blood pressure (mmHg)	135.5 +/− 11.6	121.0 +/− 5.6	<0.001
Diastolic blood pressure (mmHg)	81.35 +/− 9.4	69.7 +/− 7.8	<0.001
Fasting glucose (mg/dL)	101.4 +/− 26.0	92.6 +/− 25.0	<0.001
A1c (%)	5.5 +/− 0.8	5.3 +/− 0.5	0.134
Triglycerides (mg/dL)	149.5 +/− 63.3	133.0 +/− 52.8	<0.001
Total cholesterol (mg/dL)	201.3 +/− 49.2	195.9 +/− 45.9	0.138
HDL-cholesterol (mg/dL)	53.8 +/− 17.7	52.7 +/− 14.9	0.400

For quantitative variables, the Student´s *t*-test was used when the variables followed a Gaussian distribution. The Mann–Whitney test was used to compare non-Gaussian variables.

**Table 5:** Baseline values of gut microbiota.

	PENS-Diet (log GCN/g) (N = 30)	PENS-Diet + *L. kefiri* (log GCN/g) (N = 30)	*p* Value	Reference Range (log GCN/g)
**Muconutritive microbiota**	**6.6 +/− 1.3**	**6.9 +/− 0.9**	**0.391**	**7.0–9.0**
*Akkermansia muciniphila*	2.8 +/− 1.5	2.9 +/− 1.7	0.302	5.0–8.5
**Regulatory microbiota**	**6.6 +/− 0.9**	**6.8 +/− 0.9**	**0.380**	**6.5–8.5**
*Lactobacillus* spp.	4.6 +/− 1.4	4.9 +/− 1.1	0.367	4.5–7.0
**Proteolytic microbiota**	**7.7 +/− 1.2**	**8.3 +/− 0.9**	**0.137**	**6.5–9.0**
*Escherichia coli*	4.5 +/− 1.6	4.7 +/− 1.5	0.747	4.5–7.0
***Firmicutes phylum***	**8.2 +/− 0.9**	**8.4 +/− 0.7**	**0.381**	**8.5–11.0**
*Lactobacillus* spp.	4.6 +/− 1.4	4.9 +/− 1.1	0.367	4.5–7.0
*Faecalibacterium* spp.	6.2 +/− 1.2	6.4 +/− 0.9	0.650	7.0–9.0
*Roseburia* spp.	6.2 +/− 1.3	6.7 +/− 1.0	0.114	6.5–8.5
*Bacillus* spp.	2.0 +/− 0.9	2.0 +/− 0.7	0.962	0–4.0
*Staphylococcus* spp.	3.1 +/− 0.6	3.0 +/− 0.6	0.232	2.5–5.0
*Veillonella* spp.	4.6 +/− 0.6	4.4 +/− 0.8	0.275	4.5–7.0
*Clostridium* (*Cocc*)	7.8 +/− 1.0	8.0 +/− 0.8	0.380	7.0–9.0
*Clostridium* (*Perf*)	3.8 +/− 0.9	3.9 +/− 0.8	0.508	0–5.0
*Enterococcus* spp.	5.8 +/− 1.0	6.0 +/− 0.8	0.669	6.0–8.5
***Bacteroidetes phylum***	7.8 +/− 1.0	7.6 +/− 1.2	**0.695**	**8.0–11.0**
*Prevotella* spp.	5.7 +/− 2.0	6.0 +/− 2.2	0.470	5.0–8.5
*Bacteroides* spp.	7.3 +/− 1.1	7.1 +/− 1.3	0.617	7.5–9.0
***Firmicutes*/*Bacteroidetes***	**1.1 +/− 0.2**	**1.1 +/− 0.2**	**0.181**	**0.1–0.3**
***Proteobacteria phylum***	**5.4 +/− 1.5**	**5.3 +/− 1.3**	**0.821**	**3.0–7.0**
*Escherichia coli*	4.5 +/− 1.6	4.7 +/− 1.5	0.747	4.5–7.0
*Pseudomonas* spp.	1.7 +/− 0.9	1.5 +/− 0.7	0.296	0–4.0
*Campylobacter* spp.	1.3 +/− 1.0	1.6 +/− 1.3	0.274	0–3.5
*Helicobacter* spp.	2.2 +/− 1.1	2.1 +/− 1.1	0.738	0–3.5
***Fusobacteria phylum***	**2.8 +/− 1.2**	**3.1 +/− 1.2**	**0.508**	**0–4.5**
*Fusobacterium nucleatum*	2.8 +/− 1.2	3.1 +/− 1.2	0.508	0–4.5
***Actinobacteria phylum***	**4.3 +/− 1.7**	**4.5 +/− 1.7**	**0.801**	**6.5–9.0**
*Bifidobacterium* spp.	3.8 +/− 1.7	3.9 +/− 1.5	0.837	5.5–7.5
***Verrucomicrobia phylum***	**3.0 +/− 1.2**	**3.2 +/− 1.6**	**0.284**	**5.5–9.0**
*Akkermansia muciniphila*	2.8 +/− 1.5	2.9 +/− 1.7	0.302	5.0–8.5

For quantitative variables, the Student´s *t*-test was used when the variables followed a Gaussian distribution. The Mann–Whitney test was used to compare non-Gaussian variables.

**Table 6:** Changes in the microbiota composition in the PENS-diet group.

	Baseline (log GCN/g)	Postprocedure (log GCN/g)	*p* Value	Reference Range (log GCN/g)
**Muconutritive microbiota**	**6.6 +/− 1.3**	**6.8 +/− 1.3**	**0.06**	**7.0–9.0**
*Akkermansia muciniphila*	2.8 +/− 1.5	3.2 +/− 2.1	0.002	5.0–8.5
**Regulatory microbiota**	**6.6 +/− 0.9**	**6.6 +/− 1.4**	**0.265**	**6.5–8.5**
*Lactobacillus* spp.	4.6 +/− 1.4	5.3 +/− 1.0	0.003	4.5–7.0
**Proteolytic microbiota**	**7.7 +/− 1.2**	**8.0 +/− 1.0**	**0.065**	**6.5–9.0**
*Escherichia coli*	4.5 +/− 1.6	4.3 +/− 1.2	0.386	4.5–7.0
***Firmicutes phylum***	**8.2 +/−0.9**	**8.1 +/− 1.1**	**0.549**	**8.5–11.0**
*Lactobacillus* spp.	4.6 +/− 1.4	5.3 +/− 1.0	0.003	4.5–7.0
*Faecalibacterium* spp.	6.2 +/− 1.2	6.3 +/− 1.1	0.140	7.0–9.0
*Roseburia* spp.	6.2 +/− 1.3	6.3 +/− 1.3	0.164	6.5–8.5
*Bacillus* spp.	2.0 +/− 0.9	2.1 +/− 0.8	0.187	0–4.0
*Staphylococcus* spp.	3.1 +/− 0.6	2.9 +/− 0.5	0.140	2.5–5.0
*Veillonella* spp.	4.6 +/− 0.6	4.5 +/− 1.0	0.186	4.5–7.0
*Clostridium* (*Cocc*)	7.8 +/− 1.0	7.8 +/− 0.9	0.850	7.0–9.0
*Clostridium* (*Perf*)	3.8 +/− 0.9	4.1 +/− 1.2	0.029	0–5.0
*Enterococcus* spp.	5.8 +/− 1.0	6.0 +/− 1.8	0.117	6.0–8.5
***Bacteroidetes phylum***	7.8 +/− 1.0	8.0 +/− 1.2	**0.066**	**8.0–11.0**
*Prevotella* spp.	5.7 +/− 2.0	5.9 +/− 1.8	0.111	5.0–8.5
*Bacteroides* spp.	7.3 +/− 1.1	7.4 +/− 1.0	0.710	7.5–9.0
***Firmicutes*/*Bacteroidetes***	**1.1 +/− 0.2**	**1.0 +/− 0.1**	**0.059**	**0.1–0.3**
***Proteobacteria phylum***	**5.4 +/− 1.5**	**5.5 +/− 1.5**	**0.306**	**3.0–7.0**
*Escherichia coli*	4.5 +/− 1.6	4.3 +/− 1.2	0.386	4.5–7.0
*Pseudomonas* spp.	1.7 +/− 0.9	1.4 +/− 0.6	0.029	0–4.0
*Campylobacter* spp.	1.3 +/− 1.0	1.5 +/− 1.3	0.163	0–3.5
*Helicobacter* spp.	2.2 +/− 1.1	2.3 +/− 1.0	0.120	0–3.5
***Fusobacteria phylum***	**2.8 +/− 1.2**	**2.7 +/− 1.1**	**0.418**	**0–4.5**
*Fusobacterium nucleatum*	2.8 +/− 1.2	2.7 +/− 1.1	0.418	0–4.5
***Actinobacteria phylum***	**4.3 +/− 1.7**	**4.4 +/− 2.3**	**0.806**	**6.5–9.0**
*Bifidobacterium* spp.	3.8 +/− 1.7	3.9 +/− 2.0	0.831	5.5–7.5
***Verrucomicrobia phylum***	**3.0 +/− 1.2**	**3.5 +/− 2.3**	**0.002**	**5.5–9.0**
*Akkermansia muciniphila*	2.8 +/− 1.5	3.2 +/− 2.1	0.002	5.0–8.5

For quantitative variables, the Student´s *t*-test was used when the variables followed a Gaussian distribution. The Mann–Whitney test was used to compare non-Gaussian variables.

**Table 7:** Changes in the microbiota composition in the PENS-diet + *L. kefiri* group.

	Baseline (log GCN/g)	Postprocedure (log GCN/g)	*p* Value	Reference Range (log GCN/g)
**Muconutritive microbiota**	**6.9 +/− 0.9**	**7.6 +/− 0.9**	**0.000**	**7.0–9.0**
*Akkermansia muciniphila*	2.9 +/− 1.7	4.9 +/− 1.9	0.000	5.0–8.5
**Regulatory microbiota**	**6.8 +/− 0.9**	**7.6 +/− 1.2**	**0.000**	**6.5–8.5**
*Lactobacillus* spp.	4.9 +/− 1.1	6.2 +/− 1.3	0.000	4.5–7.0
**Proteolytic microbiota**	**8.3 +/− 0.9**	**8.4 +/− 1.1**	**0.190**	**6.5–9.0**
*Escherichia coli*	4.7 +/− 1.5	4.4 +/− 1.4	0.057	4.5–7.0
***Firmicutes phylum***	**8.4 +/− 0.7**	**8.1 +/− 0.8**	**0.011**	**8.5–11.0**
*Lactobacillus* spp.	4.9 +/− 1.1	6.2 +/− 1.3	0.000	4.5–7.0
*Faecalibacterium* spp.	6.4 +/− 0.9	6.3 +/− 1.2	0.388	7.0–9.0
*Roseburia* spp.	6.7 +/− 1.0	6.3 +/− 1.3	0.019	6.5–8.5
*Bacillus* spp.	2.0 +/− 0.7	1.8 +/− 0.6	0.201	0–4.0
*Staphylococcus* spp.	3.0 +/− 0.6	3.0 +/− 0.5	0.600	2.5–5.0
*Veillonella* spp.	4.4 +/− 0.8	4.4 +/− 0.8	0.333	4.5–7.0
*Clostridium* (*Cocc*)	8.0 +/− 0.8	8.1 +/− 0.9	0.303	7.0–9.0
*Clostridium* (*Perf*)	3.9 +/− 0.8	4.0 +/− 0.9	0.435	0–5.0
*Enterococcus* spp.	6.0 +/− 0.8	6. 2 +/− 1.0	0.372	6.0–8.5
***Bacteroidetes phylum***	7.6 +/− 1.2	9.2 +/− 1.6	0.000	**8.0–11.0**
*Prevotella* spp.	6.0 +/−2.2	7.4 +/− 2.4	0.000	5.0–8.5
*Bacteroides* spp.	7.1 +/− 1.3	7.5 +/− 1.4	0.012	7.5–9.0
***Firmicutes*/*Bacteroidetes***	**1.1 +/− 0.2**	**0.8 +/− 0.2**	**0.007**	**0.1–0.3**
***Proteobacteria phylum***	**5.3 +/− 1.3**	**4.9 +/− 1.1**	**0.022**	**3.0–7.0**
*Escherichia coli*	4.7 +/− 1.5	4.4 +/− 1.4	0.057	4.5–7.0
*Pseudomonas* spp.	1.5 +/− 0.7	1.3 +/− 0.8	0.177	0–4.0
*Campylobacter* spp.	1.6 +/− 1.3	1.3 +/− 1.0	0.095	0–3.5
*Helicobacter* spp.	2.1 +/− 1.1	1.9 +/− 0.8	0.200	0–3.5
***Fusobacteria phylum***	**3.1 +/− 1.2**	**3.0 +/− 1.2**	**0550**	**0–4.5**
*Fusobacterium nucleatum*	3.1 +/− 1.2	3.0 +/− 1.2	0.550	0–4.5
***Actinobacteria phylum***	**4.5 +/− 1.7**	**6.1 +/− 1.3**	**0.000**	**6.5–9.0**
*Bifidobacterium* spp.	3.9 +/− 1.5	5.3 +/− 1.1	0.000	5.5–7.5
***Verrucomicrobia phylum***	**3.2 +/− 1.6**	**5.2 +/− 2.0**	**0.000**	**5.5–9.0**
*Akkermansia muciniphila*	2.9 +/− 1.7	4.9 +/− 1.9	0.000	5.0–8.5

For quantitative variables, the Student´s *t*-test was used when the variables followed a Gaussian distribution. The Mann–Whitney test was used to compare non-Gaussian variables.

**Table 8:** Mean increasements of muconutritive microbiota, *Akkermansia muciniphila* and *Lactobacillus* spp. between groups.

	PENS-Diet (log GCN/g)	PENS-Diet + *L. kefiri* (log GCN/g)	*p* Value
Muconutritive microbiota	0.2 +/− 0.01	0.7 +/− 0.01	0.000
*Akkermansia muciniphila*	0.4 +/− 0.6	2.0 +/− 0.4	0.000
*Lactobacillus* spp.	0.7 +/− 0.4	1.3 +/− 0.2	0.000

For quantitative variables, the Student´s *t*-test was used when the variables followed a Gaussian distribution. The Mann–Whitney test was used to compare non-Gaussian variables.

## Data Availability

Data are unavailable due to privacy or ethical restrictions.

## References

[1] Bray G.A. (2004). Medical consequences of obesity. J. Clin. Endocrinol. Metab..

[2] Sullivan P.W., Ghushchyan V.H., Ben-Joseph R. (2008). The impact of obesity on diabetes, hyperlipidemia and hypertension in the United States. Qual. Life Res..

[3] Nguyen N.T., Magno C.P., Lane K.T., Hinojosa M.W., Lane J.S. (2008). Association of hypertension, diabetes, dyslipidemia and metabolic syndrome with obesity: Findings from the National Healcth and Nutrition Examination Survey 1999 to 2004. J. Am. Coll. Surg..

[4] Martin Duce A., del Val I.D. (2007). Cirugía de la Obesidad Mórbida.

[5] Ruiz-Tovar J., Oller I., Diez M., Zubiaga L., Arroyo A., Calpena R. (2014). Percutaneous electrical neurostimulation of dermatome T6 for appetite reduction and weight loss in morbidly obese patients. Obes. Surg..

[6] Ruiz-Tovar J., Llavero C. (2016). Long-term effect of percutaneous electrical neurostimulation of dermatome T6 for appetite reduction and weight loss in obese patients. Surg. Laparosc. Endosc. Percutaneous Tech..

[7] Ruiz-Tovar J., Llavero C., Smith W. (2017). Percutaneous electrical neurostimulation of dermatome T6 for short-term weight loss in overweight and obeses patients: Effect on ghrelin levels, glucose, lipid and hormonal profile. Surg. Laparosc. Endosc. Percutaneous Tech..

[8] Schroeder B.O. (2019). Fight them or feed them: How the intestinal mucus layer manages the gut microbiota. Gastroenterol. Rep..

[9] Lozupone C.A., Stombaugh J.I., Gordon J.I., Jansson J.K., Knight R. (2012). Diversity, stability and resilience of the human gut microbiota. Nature.

[10] Thursby E., Juge N. (2017). Introduction to the human gut microbiota. Biochem. J..

[11] Sun L., Ma L., Ma Y., Zhang F., Zhao C., Nie Y. (2018). Insights into the role of gut microbiota in obesity: Pathogenesis, mechanisms, and therapeutic perspectives. Protein Cell.

[12] Tilg H., Moschen ARKaser A. (2009). Obesity and the Microbiota. Gastroenterology.

[13] Duncan S.H., Lobley G., Holtrop G., Ince J., Johnstone A.M., Louis P., Flint H.J. (2008). Human colonic microbiota associated with diet, obesity and weight loss. Int. J. Obes..

[14] Jumpertz R., Le D.S., Turnbaugh P.J., Trinidad C., Bogardus C., Gordon J.I., Krakoff J. (2011). Energy-balance studies reveal associations between gut microbes, caloric load, and nutrient absorption in humans. Am. J. Clin. Nutr..

[15] Cerdó T., García-Santos J.A., Bermúdez M.G., Campoy C. (2019). The Role of Probiotics and Prebiotics in the Prevention and Treatment of Obesity. Nutrients.

[16] Ajala O., English P., Pinkney J. (2013). Systematic review and meta-analysis of different dietary approaches to the management of type 2 diabetes. Am. J. Clin. Nutr..

[17] Choo J.M., Leong L.E.X., Rogers G.B. (2015). Sample storage conditions significantly influence faecal microbiome profiles. Sci. Rep..

[18] Ley R.E., Hamady M., Lozupone C., Turnbaugh P.J., Ramey R.R., Bircher J.S., Schlegel M.L., Tucker T.A., Schrenzel M.D., Knight R. (2008). Evolution of mammals and their gut microbes. Science.

[19] Cole J.R. (2004). The Ribosomal Database Project (RDP-II): Sequences and tools for high-throughput rRNA analysis. Nucleic Acids Res..

[20] Dupont G., Wahl L., Alcala Dominguez T., Wong T.L., Haładaj R., Wysiadecki G., Iwanaga J., Tubbs R.S. (2020). Anatomy, physiology, and updates on the clinical management of constipation. Clin. Anat..

[21] Ruiz-Tovar J., Llavero C., Ortega I., Diez M., Zubiaga L., Calpena R. (2015). La neuroestimulación eléctrica percutánea del dermatoma T7 mejora el perfil glucémico en pacientes obesos y diabéticos tipo 2. Estudio clínico aleatorizado. Cirugía Española.

[22] Huang X., Fan X., Ying J., Chen S. (2019). Emerging trends and research foci in gastrointestinal microbiome. J. Transl. Med..

[23] Grigorescu I., Dumitrascu D. (2016). Implication of gut microbiota in diabetes mellitus and obesity. Acta Endocrinol..

[24] Roselli M., Devirgiliis C., Zinno P., Guantario B., Finamore A., Rami R., Perozzi G. (2017). Impact of supplementation with a food-derived microbial community on obesity-associated inflammation and gut microbiota composition. Genes Nutr..

[25] Montandon S.A., Jornayvaz F.R. (2017). Effects of antidiabetic drugs on gut microbiota composition. Genes.

[26] Kovatcheva-Datchary P., Nilsson A., Akrami R., Lee Y.S., De Vadder F., Arora T., Hallen A., Martens E., Björck I., Bäckhed F. (2015). Dietary fiber-induced improvement in glucose metabolism is associated with increased abundance of prevotella. Cell Metab..

[27] He F., Morita H., Hashimoto H., Hosoda M., Kurisaki J.-I., Ouwehand A.C., Isolauri E., Benno Y., Salminen S. (2002). Intestinal Bifidobacterium species induce varying cytokine production. J. Allergy Clin. Immunol..

[28] Cani P.D., Possemiers S., Van De Wiele T., Guiot Y., Everard A., Rottier O., Geurts L., Naslain D., Neyrinck A., Lambert D.M. (2009). Changes in gut microbiota control inflammation in obese mice through a mechanism involving GLP-2-driven improvement of gut permeability. Gut.

[29] Nagpal R., Wang S., Woods L.C.S., Seshie O., Chung S.T., Shively C.A., Register T.C., Craft S., McClain D.A., Yadav H. (2018). Comparative Microbiome signatures and short-chain fatty acids in mouse, rat, non-human primate, and human feces. Front. Microbiol..

[30] Lin H.V., Frassetto A., Kowalik E.J., Nawrocki A.R., Lu M.M., Kosinski J.R., Hubert J.A., Szeto D., Yao X., Forrest G. (2012). Butyrate and propionate protect against diet-induced obesity and regulate gut hormones via free fatty acid receptor 3-independent mechanisms. PLoS ONE.

[31] Geerlings S.Y., Kostopoulos I., De Vos W.M., Belzer C. (2018). Akkermansia muciniphila in the human gastrointestinal tract: When, where, and how?. Microorganisms.

[32] Everard A., Belzer C., Geurts L., Ouwerkerk J.P., Druart C., Bindels L.B., Guiot Y., Derrien M., Muccioli G.G., Delzenne N.M. (2013). Cross-talk between Akkermansia muciniphila and intestinal epithelium controls diet-induced obesity. Proc. Natl. Acad. Sci. USA.

[33] Shin N.R., Lee J.C., Lee H.Y., Kim M.S., Whon T.W., Lee M.S., Bae J.W. (2013). An increase in the Akkermansiaspp. population induced by metformin treatment improves glucose homeostasis in diet-induced obese mice. Gut.

[34] Li J., Lin S., Vanhoutte P.M., Woo C.W., Xu A. (2016). Akkermansia muciniphila protects against atherosclerosis by preventing metabolic endotoxemia-induced inflammation in Apoe-/- Mice. Circulation.

[35] Wang Z.B., Xin S.S., Ding L.N., Ding W.Y., Hou Y.L., Liu C.Q., Zhang X.D. (2019). The potential role of probiotics in controlling overweight/obesity and associated metabolic parameters in adults: A systematic review and meta-analysis. Evid. Based Complement. Altern. Med..

[36] Lorenzo O., Crespo-Yanguas M., Hang T., Lumpuy-Castillo J., Hernández A.M., Llavero C., García-Alonso M., Ruiz-Tovar J. (2020). Addition of Probiotics to Anti-Obesity Therapy by Percutaneous Electrical Stimulation of Dermatome T6.A Pilot Study. Int. J. Environ. Res. Public Health.

[37] Carasi P., Díaz M., Racedo S.M., De Antoni G., Urdaci M.C., Serradell M.d.L.A. (2014). Safety characterization and antimicrobial properties of kefir-isolated *Lactobacillus kefiri*. Biomed. Res. Int..

[38] Carasi P., Racedo S.M., Jacquot C., Romanin D.E., Serradell M.A., Urdaci M.C. (2015). Impact of kefir derived *Lactobacillus kefiri* on the mucosal immune response and gut microbiota. J. Immunol. Res..

[39] Toscano M., De Grandi R., Miniello V.L., Mattina R., Drago L. (2017). Ability of *Lactobacillus kefiri* LKF01 (DSM32079) to colonize the intestinal environment and modify the gut microbiota composition of healthy individuals. Dig. Liver Dis..

[40] Jeong D., Kim D.H., Kang I.B., Kim H., Song K.-Y., Kim H.-S., Seo K.-H. (2017). Modulation of gut microbiota and increase in fecal water content in mice induced by administration of Lactobacillus kefiranofaciens DN1. Food Funct..

